# Economic Evaluation of Inpatient Multimodal Occupational Rehabilitation vs. Outpatient Acceptance and Commitment Therapy for Sick-Listed Workers with Musculoskeletal- or Common Mental Disorders

**DOI:** 10.1007/s10926-022-10085-0

**Published:** 2023-03-23

**Authors:** Lene Aasdahl, Marius Steiro Fimland, Gudrun M.W Bjørnelv, Sigmund Østgård Gismervik, Roar Johnsen, Ottar Vasseljen, Vidar Halsteinli

**Affiliations:** 1 Department of Public Health and Nursing, Faculty of Medicine and Health Sciences, Norwegian University of Science and Technology, Trondheim, Norway; 2 Unicare Helsefort Rehabilitation Centre, Rissa, Norway; 3 Department of Physical Medicine and Rehabilitation, St. Olavs Hospital, Trondheim University Hospital, Trondheim, Norway; 4 Department of Neuromedicine and Movement Science, Faculty of Medicine and Health Science, Norwegian University of Science and Technology, Trondheim, Norway; 5 Department of Health Management and Health Economics, Faculty of Medicine, University of Oslo, Oslo, Norway; 6 Regional Centre for Health Care Development, St. Olavs Hospital, Trondheim University Hospital, Trondheim, Norway

**Keywords:** Return to work, Sick leave, Musculoskeletal diseases, Mental health, Cognitive therapy, Economic, Quality-adjusted life years

## Abstract

**Purpose:** To evaluate the cost-effectiveness and cost-benefit of inpatient multimodal occupational rehabilitation (I-MORE) compared to outpatient acceptance and commitment therapy (O-ACT) for individuals sick listed due to musculoskeletal- or common mental disorders during two-years of follow-up. **Methods:** We conducted an economic evaluation with a societal perspective alongside a randomized controlled trial with 24 months follow-up. Individuals sick listed 2 to 12 months were randomized to I-MORE (n = 85) or O-ACT (n = 79). The outcome was number of working days. Healthcare use and sick leave data were obtained by registry data. ** Results:** Total healthcare costs during the 24 months was 12,057 euros (95% CI 9,181 to 14,933) higher for I-MORE compared to O-ACT, while the difference in production loss was 14,725 euros (95% CI -1,925 to 31,375) in favour of I-MORE. A difference of 43 (95% CI -6 to 92) workdays, in favour of I-MORE, gave an incremental cost-effectiveness ratio of 278 euros for one workday, less than the cost of one day production (339 euros). Net societal benefit was 2,667 euros during two years of follow-up. **Conclusion:** Despite considerable intervention costs, the lower production loss resulted in I-MORE being cost-effective when compared to O-ACT. Based on economic arguments, I-MORE should be implemented as a treatment alternative for individuals on long-term sick leave. However, more research on subgroup effects and further follow-up of participants’ permanent disability pension awards are warranted.

**Supplementary Information:**

The online version contains supplementary material available at 10.1007/s10926-022-10085-0.

## Introduction

Negative consequences of long-term sick leave are huge both in terms of individual suffering and economic costs for companies and society [1]. Despite increasing research on identifying effective return to work (RTW) interventions, results are inconsistent, especially in terms of long-term results [2, 3]. The latter is important as sick leave spells often are recurrent.

Finding effective RTW interventions is difficult due to the complexity of long-term sick leave. This is not due to medical factors alone, but also interactions between individual, workplace, healthcare, compensation system and societal factors [4, 5]. In line with this, interventions operating across multiple domains, i.e., health, service coordination and work modification have been advocated [3]. However, these recommendations are based on only a small number of high-quality studies, and few include economic evaluations with long-term follow-up [3]. Moreover, the complex nature of interventions and different social insurance policies make comparisons across studies difficult. In a recent systematic review on RTW interventions for mental health related sick leave Dewa et al. [6] urged for more economic evaluations in different disability and health systems.

In Norway there is a long tradition for inpatient occupational rehabilitation. These programs are traditionally multimodal (consist of several components and delivered by a multidisciplinary team), transdiagnostic, group-based, and last about 4 weeks. In a recent randomized controlled trial, we evaluated the effect of 3.5 weeks inpatient multimodal occupational rehabilitation (I-MORE) for individuals sick listed due to musculoskeletal- or common mental disorders [7]. The results showed fewer days of sickness absence for I-MORE compared to a less comprehensive outpatient program consisting of acceptance and commitment therapy (O-ACT) both at 1- and 2-years follow-up [7, 8]. We hereby report on cost-effectiveness and cost-benefit of I-MORE compared to O-ACT from a societal perspective.

## Methods

### Study Design and Participants

We conducted an economic evaluation alongside a randomized clinical trial with parallel groups. The trial compared IMORE to the less comprehensive OACT for individuals on sick leave due to musculoskeletal- or common mental disorders. The primary outcome was sickness absence during 12 months of follow-up [8]. The study protocol and several other studies have been published from this project, and the description of methods is therefore partly overlapping [7; 13]. The study was approved by the Regional Committee for Medical and Health Research Ethics in Central Norway (No.: 2012/1241) and is registered in ClinicalTrials.gov (NCT01926574). The results are presented according to the CHEERS statement [14].

Eligible participants were adults 18 to 60 years who at inclusion had been sick listed 2 to 12 months with a diagnosis within the musculoskeletal (L), psychological (P) or general and unspecified (A) chapters of the ICPC-2 (International Classification of Primary Care, Second edition) [15]. Sick leave status had to be at least 50% off work at inclusion. Exclusion criteria were: (1) alcohol or drug abuse; (2) serious somatic (e.g. cancer, unstable heart disease) or psychological disorders (e.g. high suicidal risk, psychosis, ongoing manic episode); (3) specific disorders requiring specialized treatment; (4) pregnancy; (5) currently participating in another treatment or rehabilitation program; (6) insufficient oral or written Norwegian language skills to participate in group sessions and fill out questionnaires; (7) scheduled for surgery within the next 6 months; and (8) serious problems with functioning in a group setting, as assessed by the multidisciplinary clinical team.

## Interventions

Both intervention programs were developed in collaboration between health care personnel and the research group. More detailed descriptions have been published previously [8, 9]. In brief, I-MORE took place at Hysnes rehabilitation centre, which was established in 2010 as a part of St. Olavs Hospital about an hour from Trondheim, Norway. The personnel group at Hysnes consisted of physicians, psychologists, physiotherapists, and other health professions, in addition to administrative and service staff. In the study period Hysnes offered I-MORE and other rehabilitation programs [11, 16]. Hysnes had a staff capacity of treating 320 patients per year, of which 160 patients received I-MORE. This program consisted of several components: group-based Acceptance and Commitment Therapy (ACT) [17], individual and group-based physical training, mindfulness, education on various topics, and individual meetings with the coordinators in work-related problem-solving sessions, including creating an RTW-plan. The RTW- plan was sent to the general practitioner in all cases and other relevant stakeholders depending on relevance and the participants’ consent. The use and execution of the RTW-plan was up to the participant and their general practitioner. The program lasted 3.5 weeks with 6–7 h of activity each day during weekdays.

O-ACT consisted mainly of group-based ACT once a week for six weeks, each session lasting 2.5 h. The sessions were held as outpatient treatments at the Department of Physical Medicine and Rehabilitation, St. Olavs Hospital, and was led by one of two physicians or a psychologist trained in ACT. The participants were given home assignments between sessions, including a daily 15-minute audio-guided mindfulness practice. In addition, the participants were offered two individual sessions with a social worker experienced in occupational rehabilitation and trained in ACT to clarify personal values and work-related issues. The program also included a motivational group discussion with a physiotherapist on the benefits of physical training. One individual session with both the social worker and group leader present ended the program. In this session, a brief summary letter was written to the participant’s general practitioner.

### Study Context

All legal residents in Norway are included in the Norwegian public insurance system. Medically certified sick leave is compensated with 100% coverage for the first 12 months, with some limitations regarding the size of the salary. The first 16 days are covered by the employer, the rest by the Norwegian Welfare and Labour Administration. After 12 months of sick leave, it is possible to apply for more long-term medical benefits: namely work assessment allowance and permanent disability pension. Both covers approximately 66% of the income. Individuals on work assessment allowance are supposed to work according to their work capacity. Few are awarded permanent disability pension directly after 12 months and 3–4 years on work assessment allowance before application is common. Healthcare is public and universal, but not entirely free as there is an annual deductible (203 euros in 2016). Referrals to treatment are usually arranged by the general practitioners.

### Randomization and Blinding

**Fig. 1:**
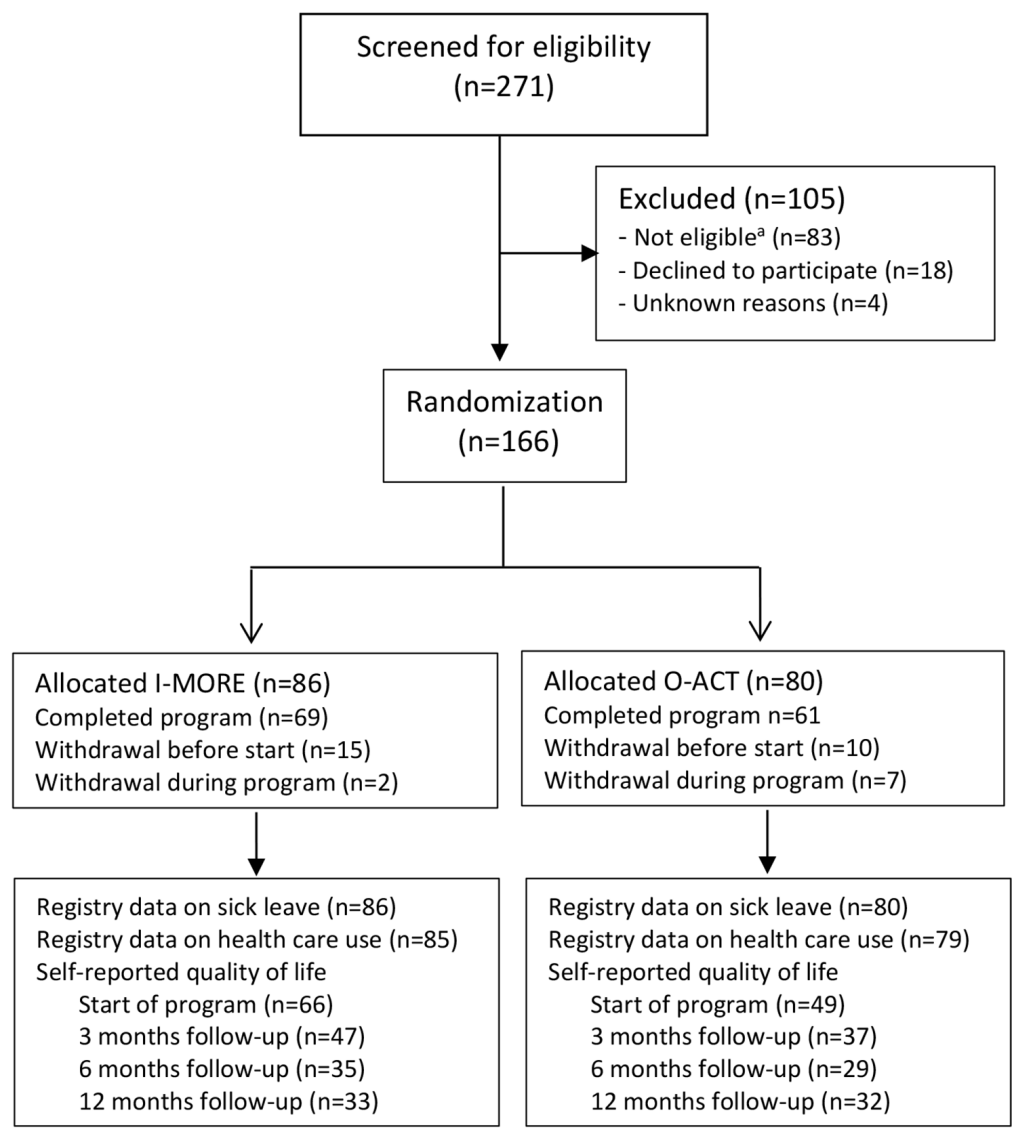
Flow of participants in the study. ^a^ Not eligible: Participating in another treatment program (n = 22), serious somatic/psychiatric illness (n = 11), specialized treatment needs (n = 4), problems with functioning in groups (n = 3), surgery scheduled next 6 months (n = 2), insufficient language skills (n = 2), alcohol/drug abuse (n = 1), no longer on sick-leave (n = 10), medical assessment not completed (n = 15), not motivated (n = 6), inability to participate in an inpatient intervention (n = 7). I-MORE: inpatient multimodal occupational rehabilitation, O-ACT: outpatient acceptance and commitment therapy

Potential participants were identified in the National Social Security System, between October 2012 and November 2014, and invited through a letter. After completing a short eligibility questionnaire, those eligible were invited for the outpatient screening assessment. If the screening was passed (Fig. 1), subjects were randomized to either I-MORE or O-ACT. A third party performed the randomization procedure (Unit of Applied Clinical Research at the Norwegian University of Science and Technology). The randomization was concealed for the researchers. It was not possible to blind neither the participants nor the caregivers for treatment. Sickness absence data was provided by the Norwegian Welfare and Labour Service, who were unaware of group allocation. The researchers were not blinded.

### Economic Evaluation

To assess costs and effects, we performed analyses of cost-effectiveness and cost-benefit. Cost-effectiveness analyses compare the cost and effect when comparing treatment options and gives an estimate of the cost per unit of effect (here workday), while cost-benefit analyses measures both costs and effects in monetary units [18]. We applied a societal perspective and estimated both healthcare costs and costs due to production loss. Total healthcare costs included intervention costs and costs of follow up during 24 months from inclusion in both the primary and secondary healthcare level. To estimate societal costs, we added the cost of production loss. All costs are presented in in 2016 euros. The average exchange rate in 2016 was 9.2899 Norwegian kroner (NOK) to one euro.

#### Intervention Costs

Intervention costs for I-MORE were estimated as a standard program cost, i.e., equal across patients, by applying the method of Time Driven Activity Costing (TDAC) [19]. First. program costs per patient were calculated by identifying the I-MORE program share of total running expenses at Hysnes rehabilitation centre according to the budget for 2013. Running expenses included wage costs for health personnel and administrative staff, commodity costs, rents, employee, and patient travel costs. Second, the therapists filled out a registration form on how their time was spent, to identify the I-MORE share of direct and indirect patient activities. Last, this information was used to allocate all time dependent types of costs from step one, such as the cost of health personnel, to an average planned I-MORE patient. Cost types that were not time dependent, such as food and over-night stay costs, were allocated according to the I-MORE share of the total inpatient days at the rehabilitation centre, and then estimated per planned I-MORE patient. The costs per patient of O-ACT were estimated with a simplified approach, dividing the total expenditures according to budget by the number of patients treated at the outpatient clinic from planned capacity in 2013. Intervention costs were adjusted to 2016-level by applying the consumer price index of Statistics Norway [20]. A detailed description of I-More and O-ACT cost calculations are presented in Online supplementary file 1.

#### Healthcare Costs Outside the Study

Information on use of healthcare services outside the study, and their related costs, were obtained from national registers. Data on the use of primary healthcare services were collected from the Norwegian Health Economics Administration and included use of general practitioner (including out-of-hour contacts), other physicians, psychologist, physiotherapy, psychomotor physiotherapy, manual therapy, chiropractor, and medical imaging. Data on the use of specialist care were collected from the Norwegian Patient Registry and included use of somatic- and psychiatric healthcare, rehabilitation, and private specialists. Data included both outpatient and inpatients visits. Within study intervention costs recorded in the registers were excluded to avoid double counting.

Valuation was performed by timing healthcare utilisation with unit costs, for example, the number of days of rehabilitation by the cost of a day of rehabilitation, to estimate the cost per participant. For primary care, the costs were estimated using the Norwegian guidelines for economic evaluations [21]. For specialist care we used information from Trondheim Municipality and the Norwegian Directorate of Health; for somatic hospital costs estimates were based on diagnosis-related groups (DRG) (Online supplementary file 2 contains descriptions). All calculations were based on 2016 tariffs.

#### Production Loss

Sick leave data were obtained from the Norwegian National Social Security System Registry, where all individuals receiving any form of medically certified sickness or disability benefits in Norway are registered by their social security number. First, we calculated the number of sickness absence days (sick leave payments, work assessment allowance or disability pension) from inclusion to 24 months follow-up. Since some patients were only partly sick-listed, we calculated the equivalent of full workdays reimbursed on medical benefits according to a 5-day workweek (full sick leave days) [7]. Costs for production loss were calculated by multiplying the number of sickness absence days in the two-year period by the average wage rate using the human capital approach [22]. The average wage rate was obtained from Statistics Norway, and estimated at 339 euros per day in 2016 [23]. The calculations were based on reported national wage (euro ~ 240) times social expenses (~ 40%). Sickness absence during the intervention period was included in the total number of sickness absence days. For the O-ACT program graded sick leave was possible, while for I-MORE this was not possible due to the inpatient setting.

#### Effect Measures

Measure of effect in the cost-effectiveness analyses was the number of working days during 24 months; working days were calculated by subtracting the number of full sick leave days from the potential number of working days in the follow-up period (522 days).

We also intended to assess quality-adjusted life years (QALYs) through cost-utility analyses, but due to large amounts of missing questionnaire data on health related quality of life [8], these analyses were dropped.

#### Questionnaire Data

In addition to registry data questionnaire data were collected at inclusion. This included anxiety and depression measured by the Hospital Anxiety and Depression Scale (HADS) [24], pain measured by one item from the Brief Pain Inventory (BPI) [25], health related quality of life measured by 15D [26] and level of education, dichotomized as high (college/university) or low.

### Statistical Analysis

For the cost-effectiveness analysis we calculated an incremental cost-effectiveness ratio (ICER) by dividing the difference in the mean costs (total health care costs) by the difference in mean number of working days during 24 months of follow-up:

$$ICER = \frac{{costs\,I{\rm{ - }}MORE - costs\,O{\rm{ - }}ACT}}{{wordays\,I{\rm{ - }}MORE - workdays\,O{\rm{ - }}ACT}} = \frac{{incremental\,costs}}{{incremental\,e\hspace{-4pt}f\hspace{-4pt}f\hspace{-4pt}ect}}$$

To avoid double counting, production loss was not included in the costs here since workdays was the effect measure (and production costs were estimated using the number of sick leave days). In the cost benefit analysis, the net societal benefit was calculated by subtracting the incremental costs (difference in total health care costs between the interventions) from the incremental benefit (difference in loss of production costs between the interventions).

For both the cost-effectiveness and the cost-benefit analyses, uncertainty was assessed using bootstrapping techniques with 1,000 repetitions. The results from the bootstraps were displayed in a cost effectiveness plane. Using the bootstrapped results, we also estimated the 95% confidence bounds as the 2.5 and 97.5 percentiles of the 1,000 bootstrapped replications.

Between group differences in total healthcare costs and for primary and secondary care were tested separately using generalized linear models with a log link and a gamma distribution [27], while the difference in sickness absence days were compared using the t-test. We also compared the difference in medians with the Mann-Whitney U test since data on sickness absence was not normally distributed.

All analyses followed intention to treat principles. A prospectively agreed analysis plan was not written, but the economic analyses were described in the published protocol [9]. All analyses were performed in STATA 17 (StataCorp. 2021. Stata Statistical Software: Release 17. College Station, TX: StataCorp LP).

## Results

Figure 1 shows the flow of participants through the study. In total, 166 participants were included and randomized to I-MORE (n = 86) or O-ACT (n = 80). Two participants, one in each arm, did not consent to linkage of data on healthcare use and were excluded. Hence, 164 (85 + 79) participants were included in the health economic evaluation. Baseline characteristics for participants in the two groups were similar (Table 1).

**Table 1:** Baseline characteristics for participants

	I-MORE (n = 85)	O-ACT (n = 79)
**Age** mean (SD)	46.2 (8.8)	45.3 (10.4)
**Women** n (%)	69 (81%)	60 (76%)
**Higher education**^a^ n (%)	31 (36%)	33 (43%)
**Work status** n (%)		
No work	11 (13%)	6 (8%)
Full time	54 (64%)	52 (66%)
Part time Graded disability pension	12 (14%) 8 (9%)	18 (23%) 3 (4%)
**Sick-leave status** ^b^ n (%)		
Full sick-leave	35 (41%)	37 (47%)
Partial sick-leave	47 (55%)	36 (46%)
Work assessment allowance	3 (4%)	6 (8%)
**Main diagnoses for sick-leave (ICPC-2)**^b^ n (%)		
A- general and unspecified	5 (6%)	9 (11%)
L- musculoskeletal	54 (63%)	39 (50%)
P- psychological	26 (31%)	31 (39%)
**Length of sick leave at inclusion** ^**b,c**^		
median days (IQR)	203 (163–264)	217 (176–268)
**Pain level**^**d**^, mean (SD)	4.9 (2.0)	4.9 (2.2)
**HADS**^**e**^ mean (SD)		
Anxiety (0–21)	7.4 (3.9)	8.6 (4.1)
Depression (0–21)	5.6 (4.1)	6.6 (3.9)
**Health related quality of life index**, mean (SD)^f^	0.61 (0.16)	0.58 (0.14)

There are some small differences from previous studies due to two persons not consenting to obtaining data on health care use and therefore not included in this paper. In addition, there are corrections and updated registry data. I-MORE: inpatient multimodal occupational rehabilitation, O-ACT: outpatient acceptance and commitment therapy. ^a^ Higher (tertiary) education (College or university). ^b^ Based on data in the medical certificate from the National Social Security System Registry. ^c^ Number of days on sick leave during the last 12 months prior to inclusion. Measured as calendar days, not adjusted for graded sick- leave or part time job. ^d^ Pain measured by one item from the Brief Pain Inventory (BPI) from 0 to 10 (0: no pain;10: worst imaginable pain). ^e^ Measured by the Hospital Anxiety and Depression Scale. ^f^ Measured by 15D at the start of rehabilitation

### Intervention and Other Healthcare Costs

The mean intervention cost of I-MORE, per patient, was 15,227 euros versus 1,188 euros for O-ACT (Table 2 and Online supplementary file 1). Participants in the two programs used similar amounts of general practitioner services in the 24-month follow-up period, while O-ACT had somewhat more use of other primary care services including medical imaging, psychologist, and physiotherapists/chiropractors (Table 2). For secondary care, the costs were higher for O-ACT for both somatic and psychiatric hospital costs, while costs due to rehabilitation and private specialist costs were similar. The difference in primary healthcare costs was 267 euros (95% CI -273 to 806, p = 0.32), while for secondary care was 1,715 euros (95% CI -960 to 4,390, p = 0.21), both in favour of I-MORE (lower costs). Total healthcare costs, excluding the intervention costs, were slightly higher for O-ACT, 6,799 vs. 4,817 euros (p = 0.17). The differences between the groups were mainly found during the first 12 months after inclusion (Online supplementary file 3).

**Table 2:** Cost per patient during 24 months of follow-up. Estimates are presented as means and standard deviation (SD) for costs in euros^a^.

	I-MORE (n = 85) Mean (SD)	O-ACT (n = 79) Mean (SD)
**Intervention program costs**	15,227	1,188
**Other healthcare costs**		
***Primary care costs***		
General practitioner^b^	1,146 (909)	1,139 (786)
Physiotherapist/chiropractor^c^	282(544)	383 (862)
Psychologist	78 (535)	147 (988)
Medical imaging	147 (183)	251 (886)
***Secondary care costs***		
Somatic hospital		
Outpatient	630 (1,096)	987 (1,560)
Inpatient	880 (2,296)	1,867 (5,953)
Psychiatric hospital Outpatient^d^	440 (1,460)	631 (2,011)
Rehabilitation		
Outpatient	63 (580)	75 (494)
Inpatient	967 (6,849)	1062 (7,319)
Private specialist	184 (712)	257 (1,066)
Total primary and secondary care	4,817 (7,626)	6,799 (10,851)
**Total health care costs**	20,044 (7,626)	7,987 (10,851)
**Production loss costs** ^e^	69,982(54,339)	84,707 (53,532)
**Sum societal costs**	90,026 (56,515)	92,693 (56,928)

I-MORE: inpatient multimodal occupational rehabilitation, O-ACT: outpatient acceptance and commitment therapy. ^a^ Converted from Norwegian Kroner to euros using 2016 numbers. ^b^ Also includes emergency primary health care service and other physicians in primary care. ^c^ Includes: Physiotherapist, manual physical therapist, psychomotor physiotherapy, and chiropractor. ^d^ Includes substance abuse care. There was no use of inpatient psychiatric health care.^e^ Calculated based on the average wage rate in 2016 obtained from Statistics Norway

### Production Costs

During 24 months of follow-up the mean number of sickness absence days were 206 (SD 160) and 250 (SD 158) for I-MORE and O-ACT, respectively, i.e., the number of working days were 316 (SD 160) for I-MORE and 272 (SD 158) for O-ACT. Consequently, the cost of production loss amounted to 69,982 euros (SD 54,339) for I-MORE and 84,707 euros (SD 53,532) for O-ACT ( p = 0.08, Table 2). See online supplement file 3 for details.

Sickness absence days were not normally distributed, and the cumulative medians were at one year for I-MORE 95 days (25th -75th percentile 44–151) vs. O-ACT 129 days (25th -75th percentile 64–213; p = 0.04), at two years: I-MORE: 161 (25th -75th percentile 61–342) vs. O-ACT 252 days (25th -75th percentile 106–380; p = 0.07).

### Cost-effectiveness Analyses

**Fig. 2:**
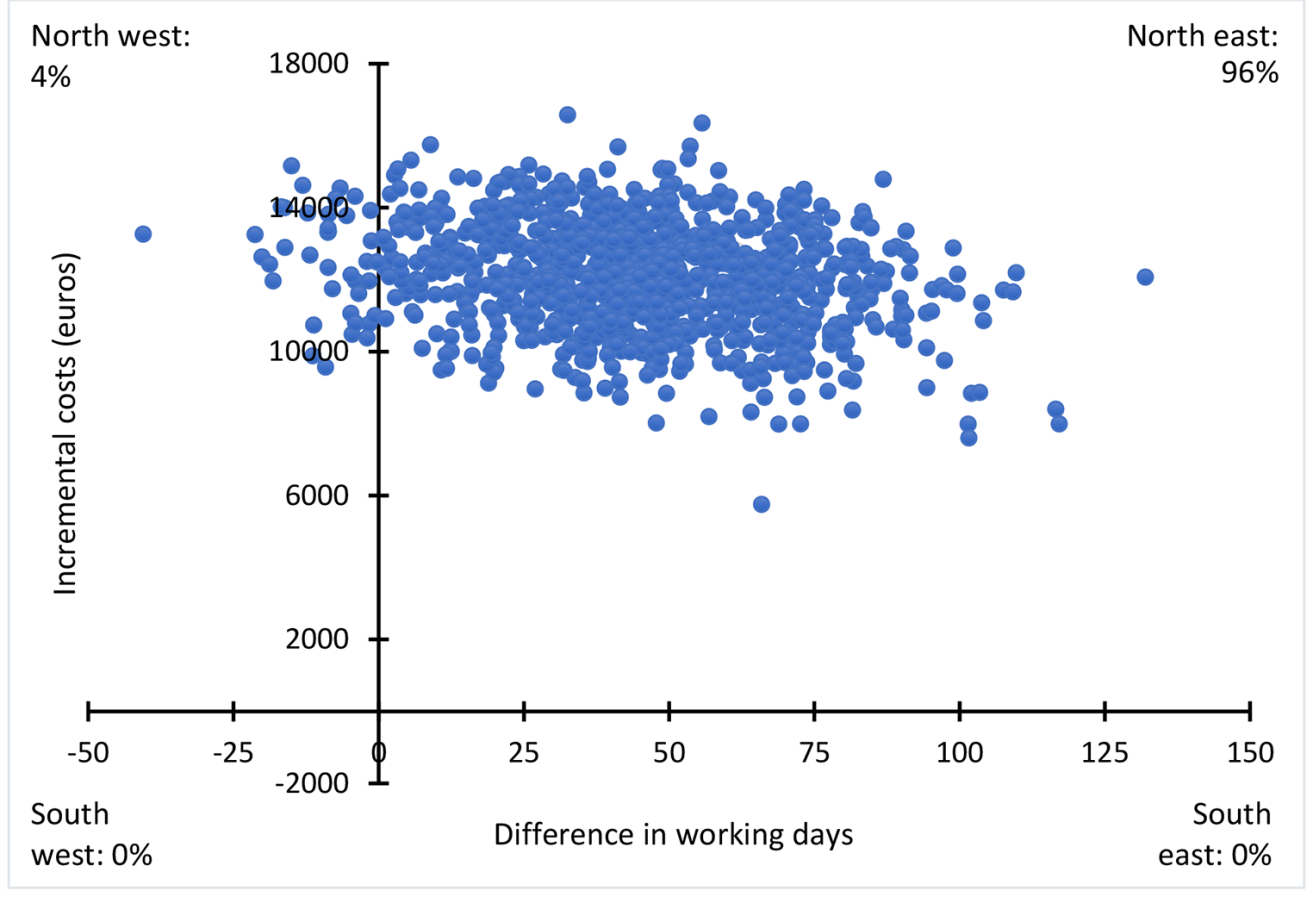
Cost effectiveness plane for difference in return to work after 24 months

Total healthcare costs were higher for I-MORE than O-ACT (Table 2). The difference after 24 months of follow-up was 12,057 euros (95% CI 9,181 to 14,933, p < 0.001). The number of workdays in the same period was however in favour of I-MORE with 43.4 days (95% CI -5.7 to 92.4), resulting in an ICER of 278 euros per workday. That is, an additional 278 euros would need to be invested in I-MORE to gain one additional day of work, when compared to O-ACT. One production day in Norway was valued to 339 euros in 2016. Figure 2 shows the cost effectiveness plane. In total, 96% of the bootstrapped cost-effect pairs (i.e., 960 of the 1,000 bootstrapped ICERs) were in the northeast corner, showing that I-MORE was more effective on RTW but also had higher costs than O-ACT; 67% of the estimates (i.e., 670 of the 1,000 bootstrapped ICERs) resulted in an ICER below 339 euros.

### Cost-benefit Analyses

Because patients in I-MORE had fewer days with sickness absence, the production loss was lower for I-MORE compared to O-ACT; during the two years of follow-up, the benefit from higher production was 14,725 euros (95% CI -1,925 to 31,375). Since the extra total health care costs (intervention costs plus other healthcare use) was 12,057 euros (95% CI 9,181 to 14,933), the net societal benefit of I-MORE was 2,667 euros in favour of I-MORE. Bootstrapped estimates showed a mean cost benefit of 2,877 euros, with confidence bounds between − 13,589 and 19,616. Among all bootstrapped estimates, 64% were above zero, thus, in favour of I-MORE.

## Discussion

I-MORE had economic benefits over O-ACT, as shown by both the cost-effectiveness, and cost-benefit approach. While intervention costs were considerably higher for I-MORE, other healthcare costs and production losses were lower when compared to O-ACT. I-MORE led to 43 fewer days of sickness absence in a 24-month perspective, even when sickness absence during the intervention period, which was inevitably higher for I-MORE, was included. Thus, when estimating the ICER, I-MORE led to an additional cost of 278 euros per additional day a patient was at work. Since 278 is less than the average wage in Norway, which was 339 euros in 2016, this resulted in a net societal benefit of 2,667 euros, in favour of I-MORE.

This is the first study performing a health economic evaluation on inpatient occupational rehabilitation in Norway. There are also few studies investigating comparable interventions internationally, especially in long-term and societal perspectives [3, 6, 28]. The results support a systematic review by Cullen et al. [3] that found strong levels of evidence for reduced lost time and costs related to work disability for work-focused cognitive behavioural therapy in mental health conditions, which was one of the components in I-MORE. They also found strong evidence for multi-domain interventions for musculoskeletal or pain-related conditions on sick leave and moderate evidence for improved costs related to work disability [3]. However, in contrast to I-MORE, all the studies with economic evaluations included in the Cullen review were workplace-based and in outpatient settings. Most countries offer occupational rehabilitation in an outpatient setting, and inpatient programs are less common. However, in Germany, work-focused inpatient rehabilitation programs compared to traditional rehabilitation, have had positive results on future RTW [29, 30].

I-MORE was considerably more expensive than O-ACT. Intervention costs for I-MORE was calculated as standard program costs per patient according to planned duration, the mix of individual and group-based activities, and according to an annual capacity. This approach was chosen since the program to a large extent was fixed and it was not considered feasible to directly observe time spent by therapists on individual patients over time. We used budget information as basis for costs, which in a Norwegian public hospital setting can be regarded as highly representative of actual expenditures. A similar method was used for O-ACT-costs, where a conservative approach was taken since no rents were included. When the results still suggest I-MORE was economically beneficial it is due to the high costs associated with production loss in O-ACT – or the benefit of more working days in I-MORE. It follows that a less conservative approach, and a potentially higher O-ACT cost will increase the economic benefits of I-MORE. Partly or graded sick leave was compiled into number of full days of sick leave in order not to overestimate the costs related to production loss [31]. Still, estimations of production loss depend highly on the price set on production loss. In the analyses we used an average wage provided by Statistics Norway [23]. The average estimates will, however, not reflect variation in the study population due to different types of employment. We also acknowledge that a lower price estimate would have changed the results. Mean cost values were used for the analyses, as is the tradition in economic evaluation. However, sickness absence was not normally distributed, and for I-MORE the median number of days of sickness absence was considerably lower than the mean. The mean estimates for production loss could therefore be assumed conservative. The difference in sickness absence days between the programs increased over time, which led I-MORE to be cost-effective in a 24-month perspective as production loss outweighed intervention costs. This underlines the importance of long-term follow-up when evaluating RTW interventions. In a previous publication [7], we showed that a larger proportion of participants in O-ACT than I-MORE transitioned to a more long-term benefit (work assessment allowance). This transition increases the chance of future permanent disability benefits, which would result in a considerably greater return on investment for I-MORE. Further research is needed to confirm this.

A substantial amount of the costs was due to the inpatient setting, meaning the intervention probably could be run at lower costs. Whether the effect of I-MORE would have been similar if it had been run as an outpatient program is an open question, but no such facilities currently exist in Norway. Advantages with an inpatient setting is that it offers a break from everyday life and an opportunity to focus on one`s own process, with regular meals and rest organized by the centre. Although overall beneficial in societal terms, providing a 3.5-week inpatient program to all potential users would potentially lead to large budget impacts on the healthcare sector. In a recent paper we found indications that I-MORE was particularly effective for individuals with insomnia [32]. Future research should identify people who particularly benefit from this type of comprehensive program. If such subgroups could be identified, the program would likely prove even more cost-effective than our results demonstrate. Furthermore, better prognostic tools would enable clinicians to offer more tailored treatment according to the individual patient`s needs.

The main strengths of this paper were the randomized design and the use of registry data for both healthcare use and sickness absence. All Norwegian citizens are registered in these registries, ensuring no missing data and no recall bias. A limitation was that production loss was based on days on sick leave without considerations of reduced productivity when at work (i.e., presenteeism). Another limitation is the lack of cost-utility analyses which we were not able to include due to a considerable amount of missing data on health-related quality of life at all measurement points. The problem of missing data is a general problem in the evaluation of RTW-programs; future studies of the cost-utility of these types of interventions are therefore warranted. Also, the effect might also vary between subgroups, like for example different diagnoses groups or educational groups, but the sample size was insufficient for subgroup analyses. In this study we used a societal perspective, where the effect was measured as absence from work/ RTW and production costs. While the findings are relevant in settings where a societal perspective is appreciated, it might not form the basis for prioritization in settings where a healthcare perspective is used.

## Conclusion

Despite high intervention costs, I-MORE was found cost-effective compared to O-ACT in a societal perspective with a two-year follow-up period. This was supported by both the cost-effectiveness and the cost-benefit analyses. We found that the costs of I-MORE was higher than the cost of O-ACT during the first year, but the effect of I-MORE on production loss during the second year outweighed the intervention costs over time. These results underscore the importance of long-term follow-up and will be of interest for both clinicians and planners of health policy.

## Electronic Supplementary Material

Below is the link to the electronic supplementary material.

Supplementary file 1: Intervention costs.

Supplementary file 2: Pricing for health care use and production loss.

Supplementary file 3: Detailed costs for health care use and production loss.

Supplementary Material 4

## Data Availability

Data is not available due to ethical approval.

## Declarations

**Authors` Contributions**.

LA, MSF, RJ, SOG and VH developed the project. MSF and RJ led the project. LA performed the analyses in collaboration with GMWB and VH. All authors (LA, MSF, GMWB, SOG, RJ, VH) critically reviewed and approved the final manuscript.
