# Supplementary material for: Economic Evaluation of Inpatient Multimodal Occupational Rehabilitation vs. Outpatient Acceptance and Commitment Therapy for Sick-Listed Workers with Musculoskeletal- or Common Mental Disorders
Source: J Occup Rehabil. 2023 Mar 23;33(3):463–72. doi: 10.1007/s10926-022-10085-0 (PMC10495483; doi:10.1007/s10926-022-10085-0)
Supplement: Supplementary file 1 — Intervention costs. [file 10926_2022_10085_MOESM1_ESM.docx]

**Supplementary file 1**

**Calculation of intervention costs per patient: I-MORE and O-ACT**

Intervention costs for inpatient multimodal occupational rehabilitation (I-MORE) and outpatient cognitive behavioural therapy (O-ACT) were estimated as standard program costs which were equal across patients. The method of time-driven activity-based costing [1] was used, and Stokkvold and Haarsaker have provided a detailed description of the methods, data and results in their master thesis from 2013 [2]. The two main data sources were expenditures according to budgets and information of time spent by therapists.

Hysnes Rehabilitation Centre (HRC) was established in 2010 and 2013 was the first year with a complete twelve-months provision of rehabilitation services, including I-MORE. Since the annual account information for 2013 was yet not available, cost calculations were based on available budget for HRC. The same applies to the outpatient clinic of the Department of Physical Medicine and Rehabilitation (DPMR), where O-ACT was offered. Table 1 shows the total budget for HRC and the outpatient clinic, and the relative distribution on cost components. Costs were adjusted to 2016-level by applying the consumer price index of Statistics Norway [3]. The average exchange rate in 2016 was 9.2899 Norwegian kroner (NOK) to one euro.

*Table 1: Budget for the year of 2013 for Hysnes Rehabilitation Centre and the outpatient clinic of the Department of Physical Medicine and Rehabilitation. All costs in 2016 euros.*

|  | Hysnes Rehabilitation Centre | | Outpatient clinic at the Dep. of Physical Medicine and Rehabilitation | |
| --- | --- | --- | --- | --- |
|  | 1000 euro | % | 1000 euro | % |
| Salaries | 2, 268 | 54 % | 122 | 86 % |
| Commodities | 329 | 8 % | - | 0 % |
| Rents | 948 | 23 % | - | 0 % |
| Travel | 78 | 2% | - | 0 % |
| Other costs | 550 | 13 % | 20 | 14 % |
| **Total budget** | **4, 173** | **100 %** | **142** | **100%** |

The budget for running expenses at HRC included all relevant costs:

- Salaries: wage costs for health personnel and administrative staff
- Commodities: Medical supplies, food supplies
- Rents: Rent costs and caretaking
- Travel: Travel costs for patients and employees
- Other cost: Power, office equipment, amortisation (capital consumption)

The budget for HRC included total costs for the centre, and the total planned capacity was 320 patients per year. I-MORE was one of three treatment programs provided, each with a planned capacity of 160 patients. For the description of how costs were allocated to I-MORE, see below. Table 1 shows that wage costs for HRC in total accounted for 54 % of planned expenditures, while housing related costs including food supplies amounted to 31%. Travel costs was reimbursed for both patients and employees (2%), the latter because most employees were commuting from the closest city (Trondheim) to the rural Hysnes.

The budget for the outpatient clinic at the DPMR included the provision of O-ACT only and the planned capacity was 120 patients per year. Therapists working at the DPMR were employed both at the outpatient clinic and at other sections and the budget for the outpatient section included the cost of the outpatient share of the positions only. Wage costs accounted for 86 % of the expenditures. Rent costs were not included and compared to the costs included for HRC this implies that a conservative approach was taken. One can argue that O-ACT costs were slightly underestimated.

To identify the I-MORE cost share of total HRC costs, all therapists were asked to fill out a questionnaire covering the time spent on direct and indirect patient activities for the three different treatment programs respectively:

- Ordinary program I
- Ordinary program II
- I-MORE

The questionnaire covered two representative weeks in February 2013 and table 2 shows a summary of the findings. Time spent by therapists for the O-ACT program was based on an interview with the head of the outpatient clinic.

All the rehabilitation programs were planned programs, in the sense that duration (number of days) and the distribution between group sessions versus individual follow-up activities for therapists to a large extent was fixed. The survey identified that 29% per cent of therapist time was allocated to I-MORE.

*Table 2: Registration of time spent by therapists on the three different programs.*

|  | Hysnes Rehabilitation Centre | | | | Outpatient clinic at the Dep. of Phys. Medicine and Rehabilitation |
| --- | --- | --- | --- | --- | --- |
|  | Ordinary program (I) (not included in study) | Ordinary program (II) (not included in study) | **Study program I-MORE** | SUM – all programs | **Study program**  **O-ACT** |
| With patient (group or individually) | 28% | 15% | **21%** | 23 % | **25 %** |
| External activity (NAV, GP, employer) | 1% | 1% | **0%** | 1% | **0 %** |
| Administrative tasks (meetings, journal writing, travel etc.) | 71% | 84% | **79%** | 76 % | **75 %** |
| Sum | 100% | 100% | **100%** | 100 % | **100%** |
| Distribution of time between programs | 47% | 24% | **29%** | 100% |  |

NAV: National Social Security System office; GP: general practitioner

By applying the method of time-driven activity-based costing [1], the I-MORE share of therapist time was used to allocate relevant therapist costs like wage and travel per I-MORE patients. For all other costs the I-MORE share of total patient days staying at HRC, according to planned capacity, was used to allocate costs (commodities, rents, and other costs). The result was an estimated cost per patient of 15,227 euros.

The cost per patient of O-ACT was calculated by taking the total expenditure from table 1 divided by number of patients, according to planned capacity in 2013. The result was an estimated cost per patient of 1,188 euros.

**References:**

1. Kaplan RS, Anderson SR. Time-driven activity-based costing: a simpler and more powerful path to higher profits. Harvard business press; 2007.

2. Stokkvold K.M.; Haarsaker T. Kostnadskalkyler i helsesektoren [Cost calcualations in health care] [Master`s thesis]. Trondheim, Norway: NTNU; 2013.

3. Statistics Norway. Inflation calculator. <https://www.ssb.no/kalkulatorer/priskalkulator>. Accessed April 21st 2022.
