# Supplementary material for: Economic Evaluation of Inpatient Multimodal Occupational Rehabilitation vs. Outpatient Acceptance and Commitment Therapy for Sick-Listed Workers with Musculoskeletal- or Common Mental Disorders
Source: J Occup Rehabil. 2023 Mar 23;33(3):463–72. doi: 10.1007/s10926-022-10085-0 (PMC10495483; doi:10.1007/s10926-022-10085-0)
Supplement: Supplementary file 2 — Pricing for health care use and production loss. [file 10926_2022_10085_MOESM2_ESM.docx]

**Supplementary file 2**

**Table 1** Pricing for different types of health care use and production loss. Prices are in euros (EUR)^a^

| **Category** | **Unit cost** | **Source of information** |
| --- | --- | --- |
| **Primary care** |  |  |
| General practitioner^b^ | Depending on consultation  Median 14 EUR (min 0; max 689)  Calculated as reimbursement^c^ to physician x2 | Guidelines^e^ |
| Psychologist | Depending on consultation  Median 107 EUR (min 38; max 2,022)  Calculated as reimbursement^c^ to psychologists x2 | Guidelines^e^ |
| Medical imaging | Depending on type  Median 87 EUR (min 4; max 7,328)  Calculated as (reimbursement^c^+ individual share paid by Health Economics Administration+ share paid by the patient) x2 | Guidelines^e^ |
| Physiotherapist/chiropractor^d^ | Depending on treatment  Median 27 EUR (min 3; max 104)  Calculated as reimbursement^c^+ individual share paid by Health Economics Administration+ share paid by the patient | Guidelines^e^ |
|  |  |  |
| **Specialist health care** |  |  |
| Somatic hospital | Diagnosis based costs (DRG) weighted by complexity, type of visit/stay and treatment. Pricing updated annually^f^.  Median 136 EUR (min 18; max 22,096) | The Norwegian Directorate of Health |
| Psychiatric hospital  Outpatient care | 218 EUR | The Norwegian Directorate of Health |
| Substance abuse clinic | 320 EUR | The Norwegian Directorate of Health |
| Rehabilitation | Inpatient 411 EUR (per day)  Outpatient 206 EUR (inpatient divided by two) | Trondheim municipality |
| Private specialist physician | 119 EUR | Guidelines^e^ |
|  |  |  |
| **Production loss** | 339 EUR (per workday)^g^ | Statistics Norway |
|  |  |  |

^a^ Converted from Norwegian Kroner to euros using 2016 numbers.

^b^ Also includes emergency primary health care service and other physicians in primary care.

^c^ Reimbursements to the clinician from The Norwegian Health Economics Administration

^d^ Includes: Physiotherapist, manual physical therapist, psychomotor physiotherapy, and chiropractor.

^e^ Guidelines: Norwegian guidelines for economical evaluations of health measures, published by The Norwegian Directorate of Health

^f^ If diagnoses-based costs were missing for 2016, the closest year available was used instead.

^g^ Includes salary (245 EUR) and social costs (factor 1.3859): 245*1.3859=339.
