# Supplementary material for: Economic Evaluation of Inpatient Multimodal Occupational Rehabilitation vs. Outpatient Acceptance and Commitment Therapy for Sick-Listed Workers with Musculoskeletal- or Common Mental Disorders
Source: J Occup Rehabil. 2023 Mar 23;33(3):463–72. doi: 10.1007/s10926-022-10085-0 (PMC10495483; doi:10.1007/s10926-022-10085-0)
Supplement: Supplementary file 3 — Detailed costs for health care use and production loss. [file 10926_2022_10085_MOESM3_ESM.docx]

**Supplementary file 3**

**Table 1** Detailed costs for health care use and production loss for 24 months of follow-up. Estimates are presented as means and standard deviation (SD) for costs in euros^a^.

|  | **I-MORE**  (n=85)  Mean (SD) | | **O-ACT**  (n=79)  Mean (SD) | |
| --- | --- | --- | --- | --- |
|  | Number of visits  Mean (SD) | Costs  Mean (SD) | Number of visits  Mean (SD) | Costs  Mean (SD) |
| **Primary care** |  |  |  |  |
| General practitioner^b^  0-4 months  5-8 months  9-12 months  13-24 months | 5.8 (3.1)  4.7 (4.2)  4.0 (4.3)  10.8 (7.7) | 238 (173)  225 (241)  193 (285)  490 (623) | 6.5 (3.8)  5.0 (4.1)  3.7 (3.3)  11.4 (8.2) | 304 (272)  248 (216)  161 (183)  425 (374) |
| Physiotherapist/chiropractor^c^ 0-4 months  5-8 months  9-12 months  13-24 months | 1.0 (2.3)  1.1 (2.4)  1.8 (4.4)  5.7 (12.2) | 26 (66)  21 (55)  53 (160)  183 (439) | 2.7 (6.4)  2.2 (5.4)  2.0 (5.2)  5.8 (13.3) | 92 (234)  69 (179)  50 (143)  172 (459) |
| Psychologist  0-4 months  5-8 months  9-12 months  13-24 months | 0  0  0  0.4 (3.3) | 0  0  0  78 (535) | 0.2 (1.6)  0.2 (1.5)  0.2 (1.5)  0.8 (5.2) | 21 (184)  18 (156)  22 (141)  87 (540) |
| Medical imaging  0-4 months  5-8 months  9-12 months  13-24 months | 0.3 (0.7)  0.2 (0.5)  0.2 (0.5)  0.7 (0.9) | 31 (81)  25 (65)  18 (62)  73 (122) | 0.4 (0.8)  0.3 (0.9)  0.3 (0.6)  0.7 (1.3) | 135 (843)  32 (91)  23 (67)  62 (125) |
|  |  |  |  |  |
| **Secondary care** |  |  |  |  |
| Somatic hospital visit  Outpatient  0-4 months  5-8 months  9-12 months  13-24 months  Inpatient  0-4 months  5-8 months  9-12 months  13-24 months | 0.8 (2.2)  0.7 (2.6)  0.7 (2.0)  1.7 (3.9)  0  0.1 (0.6)  0.3 (1.4)  0.3 (1.2) | 154 (542)  100 (305)  120 (319)  256 (490)  0  153 (678)  346 (1,470)  381 (1,279) | 1.1 (3.1)  1.3 (3.5)  0.8 (1.9)  2.8 (7.9)  0.1 (0.4)  0.03 (0.2)  0.2 (0.8)  0.7 (3.6) | 264 (1008)  253 (760)  108 (261)  362 (730)  231 (940)  79 (647)  186 (796)  1,370 (5,677) |
| Psychiatric hospital Outpatient visit^c^  0-4 months  5-8 months  9-12 months  13-24 months  Substance abuse clinic  0-4 months  5-8 months  9-12 months  13-24 months | 0.3 (1.1)  0.4 (1.7)  0.5 (1.8)  0.9 (3.7)  0.01 (0.11)  0.01 (0.11)  0  0 | 54 (249)  79 (358)  97 (381)  202 (812)  4 (35)  4 (35)  0  0 | 0.9 (3.0)  0.8 (2.5)  0.5 (2.5)  0.7 (3.2)  0  0  0  0 | 190 (660)  162 (547)  118 (543)  160 (696)  0  0  0  0 |
| Rehabilitation  Outpatient  0-4 months  5-8 months  9-12 months  13-24 months  Inpatient  0-4 months  5-8 months  9-12 months  13-24 months | 0  0  0  0.3 (2.8)  0  0  1.8 (16.3)  0.6 (3.9) | 0  0  0  63 (580)  0  0  725 (6,689)  242(1,588) | 0  0  0  0.4 (2.4)  2.0 (17.4)  0  0.3 (2.4)  0.4 (3.2) | 0  0  0  75 (494)  807 (7,169)  0  109 (971)  146 (1,295) |
| Private specialist  0-4 months  5-8 months  9-12 months  13-24 months | 0.1 (0.3)  0.3 (2.2)  0.4 (2.6)  0.7 (3.4) | 15 (40)  36 (259)  49 (311)  84 (408) | 0.4 (1.7)  0.4 (1.6)  0.4 (1.6)  1.1 (4.6) | 44 (199)  42 (190)  47 (189)  125 (550) |
|  |  |  |  |  |
|  | **Mean number of days (SD)** | **Costs^a^**  **Mean (SD)** | **Mean number of days**  **(SD)** | **Costs^a^**  **Mean (SD)** |
| **Sick leave** |  |  |  |  |
| 0-4 months  5-8 months  9-12 months  13-24 months | 48.6 (24.5)  31.0 (29.7)  29.7 (31.0)  96.9 (91.5) | 16,498 (8,332)  10,515 (10,075)  10,090 (10,538)  32,879 (31,055) | 52.3 (27.4)  39.6 (28.3)  41.5 (32.6)  116.1 (91.7) | 17,748 (9,305)  13,456 (9,615)  14,083 (11,063)  39,419 (31,118) |

I‑MORE: inpatient multimodal occupational rehabilitation, O‑ACT: outpatient acceptance and commitment therapy

^a^ Converted from Norwegian Kroner to euros using 2016 numbers

^b^ Also includes emergency primary health care service and other physicians in primary care

^c^ Includes: Physiotherapist, manual physical therapist, psychomotor physiotherapy, and chiropractor.

^d^ There were no inpatient use
